# The proprotein convertase PC1/3 regulates TLR9 trafficking and the associated signaling pathways

**DOI:** 10.1038/srep19360

**Published:** 2016-01-18

**Authors:** M. Duhamel, F. Rodet, A. N. Murgoci, R. Desjardins, H. Gagnon, M. Wisztorski, I. Fournier, R. Day, M. Salzet

**Affiliations:** 1 Univ. Lille, INSERM, U1192 - Laboratoire Protéomique, Réponse Inflammatoire et Spectrométrie de Masse-PRISM, F-59000 Lille, France; 2 Institut de Pharmacologie, Département de Chirurgie/Service d’Urologie, Faculté de Médecine et des Sciences de la Santé, Université de Sherbrooke, 3001 12^e^ Ave Nord, Sherbrooke, Qc, Canada, J1H 5N4

## Abstract

Endosomal TLR9 is considered as a potent anti-tumoral therapeutic target. Therefore, it is crucial to decipher the mechanisms controlling its trafficking since it determines TLR9 activation and signalling. At present, the scarcity of molecular information regarding the control of this trafficking and signalling is noticeable. We have recently demonstrated that in macrophages, proprotein convertase 1/3 (PC1/3) is a key regulator of TLR4 Myd88-dependent signalling. In the present study, we established that PC1/3 also regulates the endosomal TLR9. Under CpG-ODN challenge, we found that PC1/3 traffics rapidly to co-localize with TLR9 in CpG-ODN-containing endosomes with acidic pH. In PC1/3 knockdown macrophages, compartmentalization of TLR9 was altered and TLR9 clustered in multivesicular bodies (MVB) as demonstrated by co-localization with Rab7. This demonstrates that PC1/3 controls TLR9 trafficking. This clustering of TLR9 in MVB dampened the anti-inflammatory STAT3 signalling pathway while it promoted the pro-inflammatory NF-kB pathway. As a result, macrophages from PC1/3 KO mice and rat PC1/3-KD NR8383 macrophages secreted more pro-inflammatory cytokines such as TNF-α, IL6, IL1α and CXCL2. This is indicative of a M1 pro-inflammatory phenotype. Therefore, PC1/3 KD macrophages represent a relevant mean for cell therapy as “Trojan” macrophages.

During tumorigenesis, key immune cells such as macrophages are recruited to the tumor site to become tumour associated macrophages (TAMs). However, the tumor creates an immune suppressive environment, which orients TAMs toward the anti-inflammatory M2 phenotype. In turn, these TAMs suppress immune cells function by secreting anti-inflammatory molecules and lack secretion of pro inflammatory immune components. Presence of TAMs has been closely correlated with vascularisation, metastasis and resistance to immunotherapy leading to a poor prognosis and outcome in most types of cancer^1^. Skewing TAMs towards pro-inflammatory phenotype is therefore extensively investigated as potential means for developing novel antitumor therapy. Such a strategy to reactivate TAMs consists in activating key innate immune receptors such as Toll-like receptors. In this context, the endosomal TLR9 is considered as a potent therapeutic target. It is involved in the recognition of double-stranded DNA rich in unmethylated CpG motifs of bacterial or viral origin^2^,^3^. At present, its synthetic ligand CpG ODN is currently tested as cancer vaccine adjuvant^4^. Indeed, intra-tumoral injection of CpG-ODN reduced the frequency of regulatory T cells (Tregs)^4^ and decreased the number and suppressive activity of tumor infiltrating monocyte-derived suppressor cells (MDSC)^5^. These MDSC express TLR9 and respond to CpG-ODN stimulation by (i) losing their ability to suppress T cell function, (ii) producing Th1 cytokines and (iii) differentiating into tumoricidal macrophages. CpG-ODN delivery in the tumors also promoted cytotoxic responses by recruiting and inducing CD8 T cells^6^,^7^. Unexpectedly, intra-tumoral injection of CpG-ODN contributed to partial or complete remission of various tumors in humans^8^ and rodents^7^.

In our opinion, combining CpG-ODN intra-tumoral delivery and cell therapy with macrophages highly responsive to CpG-ODN is a promising strategy to treat tumors more efficiently. To obtain these highly reactive macrophages, it is crucial to decipher the mechanisms controlling TLR9 activation and signalling. TLR9 activation and signalling are intimately linked to TLR9 localization and trafficking. Indeed, recognition of CpG DNA by TLR9 is accompanied by changes in membrane dynamics and trafficking, resulting in their strict spatiotemporal compartmentalization^9^. During the resting state, TLR9 is synthesized in the endoplasmic reticulum (ER)^10^. After bacterial or viral infection, it translocates to the endolysosomal system to interact with its ligand and triggers associated signaling pathways. Nevertheless, apart from UNC93B1^11^, PRAT4^12^, HMGB1^13^, GP96^14^, protein kinase D1^15^ and Ly49Q^16^, the factors regulating TLR9 trafficking remain largely unknown.

We have recently demonstrated that proprotein convertase 1/3 (PC1/3) inhibition in macrophages led to marked perturbation of intracellular trafficking machinery through dysregulation of cytoskeletal protein expression. As a consequence, biogenesis of endosome carrier vesicle (ECV)/multivesicular body (MVB) transport intermediates from early endosomes was modified. Proteins involved in the late steps of the endosomal MVB pathway were also over-expressed. We have also pointed out that TLR4-Myd88 dependent signalling was exacerbated via the autocrine pathway. This promoted development of a pro-inflammatory M1-like phenotype in the presence or absence of endotoxin challenge^17^. In the present study, we wished to address if PC1/3 may also control the activity of the endosomal TLR9. We first demonstrated that macrophages from PC1/3-KO mice treated with CpG-ODN synthesized and secreted increased amounts of the pro-inflammatory cytokines TNF-α and IL-6. Since TLR9 signalling and the resulting cytokine secretion depend of TLR9 localization, we investigated the impact of PC1/3 inhibition on TLR9 dynamics and trafficking. For this purpose, we used the rat macrophage pulmonary cell line NR8383. Indeed, we have previously shown that it is a useful cell line to study the link between PC1/3 and regulation of cytokine secretion^18^,^19^. We found that PC1/3 travelled rapidly to co-localize with TLR9 in CpG-ODN-containing endosomes with acidic pH. Moreover, in PC1/3 knockdown (KD) cells, compartmentalization of TLR9 was altered and TLR9 formed clusters in multivesicular bodies. As a consequence, the NF-κB pro-inflammatory pathway was more rapidly and strongly activated whereas the STAT3 anti-inflammatory pathway was repressed. Because TLR9, NF-κB and STAT3 are key molecules involved in tumor propagation^20^,^21^, control of their activation by PC1/3 represents a promising and potent antitumoral therapy. Therefore, PC1/3 KD macrophages represent a relevant mean for cell therapy as “Trojan” macrophages.

## Results

### Peritoneal macrophages isolated from PC1/3 KO mice exhibit excessive TNFα and IL-6 production and secretion

**Figure 1:**
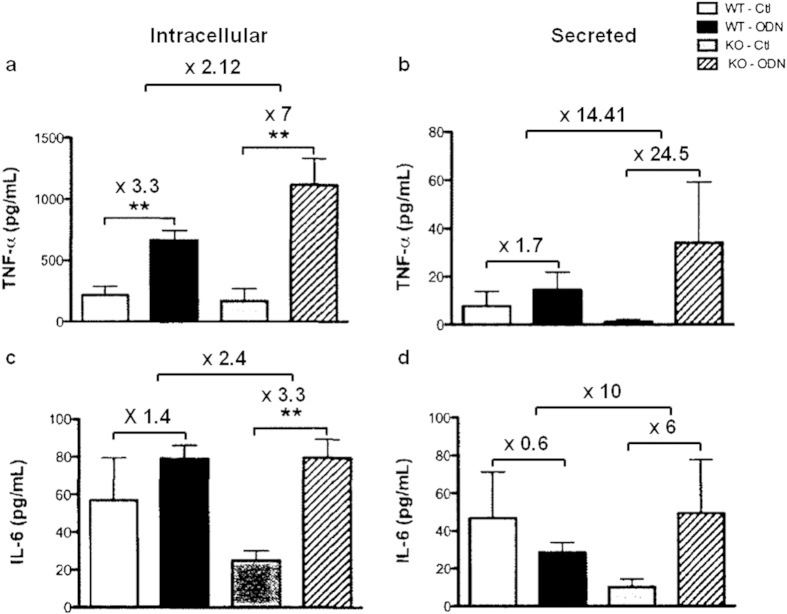
TNFα and IL-6 synthesis and secretion from peritoneal macrophages isolated from PC1/3 KO mice under CpG-ODN challenge. Under CpG-ODN challenge, peritoneal macrophages isolated from PC1/3 KO mice synthesize (**a**) and secrete (**b**) more TNFα than those harvested from WT mice. Similar results were obtained for IL-6 production (**c**) and secretion (**d**). Peritoneal macrophages were stimulated for 4 h with 1 μM CpG-ODN 2006 and TNFα or IL-6 concentration was measured using an ELISA kit. The data represent the mean ± SE. (n = 4; *p < 0.05; **p < 0.01 as determined by the t-test).

We have previously shown that PC1/3 regulated cytokine secretion in mice challenged with LPS^18^. Indeed, the plasma levels of proinflammatory cytokines (IL-6, IL-1β, and TNFα) were very significantly elevated in PC1/3 KO mice injected intraperitoneally with LPS. Peritoneal macrophages isolated from these mice and stimulated with this endotoxin also displayed uncontrolled cytokine secretion. To determine whether PC1/3 also controls signaling pathways and cytokine secretion associated with an endosomal TLR, such as TLR9, peritoneal macrophages were isolated from PC1/3 KO mice and challenged for 4 h with 1 μM CpG-ODN 2006 (Fig. 1). Following CpG-ODN stimulation, both WT and PC1/3 KO macrophages clearly produced (Fig. 1a) and secreted (Fig. 1b) an increased amount of TNFα. Comparison of the fold changes in cytokine levels between the stimulated and control cells revealed that the fold changes in TNFα synthesis (Fig. 1a) and release (Fig. 1b) were 2.12- and 14.41-fold higher, respectively, in the PC1/3 KO macrophages. Similar results were obtained for IL-6 production (Fig. 1c) and secretion (Fig. 1d) with a synthesis and release of IL-6, respectively 2.5- and 10-fold higher in PC1/3 KO macrophages. These findings demonstrate that PC1/3 has inhibitory effects on pro-inflammatory cytokine production and secretion induced by challenge with 1 μM CpG-ODN 2006. Thus, PC1/3 may regulate TLR9 signalling and the resulting cytokine secretion. The NR8383 rat pulmonary macrophage cell line is a cellular model used to study innate immunity. It also presents similar pro protein convertase expression as macrophages isolated from mice. Indeed, this cell line has been previously shown to be a good model for studying the role of PC1/3 in the macrophage innate immune response^18^,^19^. Therefore, we chose to use these cells to characterize the molecular and cellular mechanisms controlled by PC1/3. First, the effect of CpG-ODN treatment on PC1/3 activation was tested.

### CpG-ODN induces PC1/3 cleavage and activation

**Figure 2:**
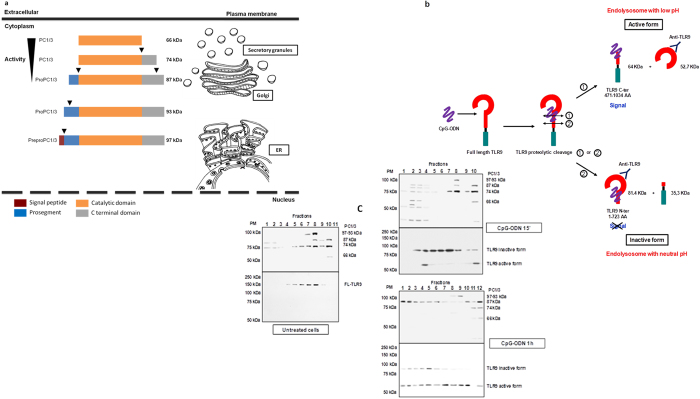
PC1/3 and TLR9 activation under CpG-ODN treatment. (**a**) Schematic representations of PC1/3 forms (realized by dr. F. Rodet). Cleavage sites are indicated by the black arrows. The apparent molecular weights of the various PC1/3 forms are indicated to the right of each bar. (**b**) Schematic representations of TLR9 cleavage forms. The antibody is directed against the extracellular domain of TLR9. The molecular weights of the various forms recognized by this antibody after proteolytic cleavage are indicated. (**c**) Subcellular fractionation of NR8383 cells. Cells were left untreated or stimulated with 1 μM CpG-ODN 2006 for 15 min or for 1 h and were then subjected to gradient fractionation. Next, aliquots of each collected gradient fraction were processed using SDS-PAGE (8%) and were immunoblotted with antibodies directed against PC1/3 and TLR9.

PC1/3 activation requires specific proteolytic cleavage, which occurs during its trafficking from the endoplasmic reticulum (ER) to secretory granules (Fig. 2a)^22^. Five molecular forms of PC1/3 have been described to date. The 97 kDa form corresponds to the preproprotein. It is synthesized in the ER and rapidly cleaved to give rise to the 93 kDa proprotein, which traffics between the endoplasmic reticulum (ER) and the TGN. In the TGN, it is processed to generate full-length (87 kDa) PC1/3. A C-terminal truncated active 74 kDa form is then produced and transported to secretory granules, where it is processed into the full active 66 kDa form. TLR9 activation is also directly linked to its spatiotemporal compartmentalization and specific proteolytic cleavage (Fig. 2b). Following its trafficking to specific endolysosomes, it is cleaved to give rise either to its active or inactive form^23^. In endolysosomes with a low pH, the N-terminus of TLR9 is processed to generate a 64 kDa C-terminal fragment. TLR9 with this fragment is referred to as the active form because this fragment recruits MyD88 and induces signaling when it binds CpG-ODN. In contrast, in endolysosomes with a neutral pH, cleavage occurs in the C-terminus of the receptor to produce an 81.4 kDa N-terminal fragment. Despite its binding to CpG-ODN, this fragment cannot trigger any signaling since it lacks a TIR domain. It is thus considered as the inactive form of TLR9.

Therefore, we wished to determine whether there was a correlation between the active or inactive form of TLR9 and the active form of PC1/3. For this purpose, we performed subcellular fractionation using Optiprep™ density gradient centrifugation and western blotting with antibodies directed against PC1/3 and TLR9 (Fig. 2c). In untreated cells, we detected the various steady-state forms of PC1/3 and only unprocessed TLR9. After 15 min of CpG-ODN stimulation, a portion of TLR9 was detected in a PC1/3-positive compartment (Fig. 2b). At this time point, cleaved forms of TLR9 appeared, the majority of which were inactive. PC1/3 is also processed for activation, as reflected by detection of more of the active 74 kDa and 66 kDa forms (fractions 2 and 3). The 66 kDa form is typically weakly detected due to its natural instability^22^. Accordingly, after 1 h of stimulation, this form as well as the C-terminal truncated active 74 kDa form disappeared. In contrast, an increase in the 87 kDa form was observed. These findings reflect the synthesis and trafficking of PC1/3 from the endoplasmic reticulum (ER) to the TGN to be processed and to produce a sufficient amount of active PC1/3. Interestingly, the majority of TLR9 was found to be active in all fractions except 11. This finding suggests that PC1/3 may control TLR9 activation. To test this hypothesis, we first examined the effect of CpG-ODN treatment on PC1/3 intracellular localization.

### CpG-ODN induces PC1/3 translocation in endosomes

**Figure 3:**
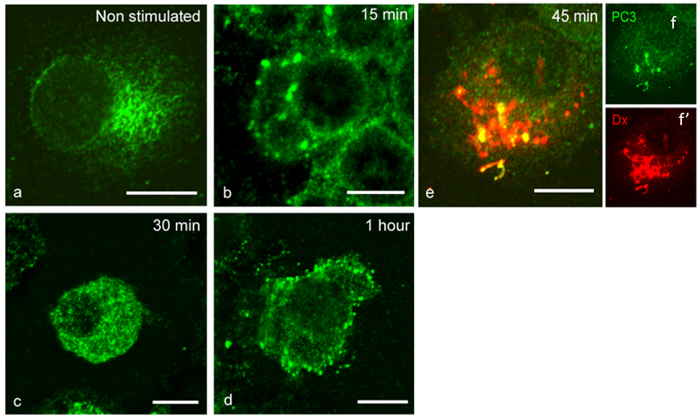
PC1/3 trafficking is affected by CpG-ODN internalization. PC1/3 was labelled by indirect immunofluorescence using anti-PC1/3 (green, Alexa488) and imaged by confocal microscopy. (**a**) Non-stimulated NR8383 cells. (**b–e**) NR8383 cells incubated with 100 nM CpG-ODN 2006 for the indicated durations. (**e**) Co-localization of PC1/3 (green, Alexa488) and Texas Red labelled dextran identified PC1/3-positive vesicular structures as lysosomes (big) and endosomes (small). Bars = 10 μm.

We have previously demonstrated that in resting NR8383 cells, PC1/3 is localized to the Golgi apparatus^19^. In the present study, we performed confocal microscopy (Fig. 3a) and showed that CpG-ODN treatment induced PC1/3 trafficking (Fig. 3b–d). After only 15 min of exposure to CpG-ODN, many vesicular structures containing PC1/3 began to appear (Fig. 3b). By 30 min of incubation, almost the whole cytoplasm was filled with these vesicular structures (Fig. 3c). After 1 h of stimulation, PC1/3-containing vesicular structures could be observed migrating toward the cell periphery and plasma membrane (Fig. 3d). Co-localization studies of PC1/3 and fluorescently labelled dextran identified these structures as endolysosomal compartments (Fig. 3e).

### PC1/3 and TLR9 traffic and co-localize to the same acidic endosomes under CpG-ODN treatment

**Figure 4:**
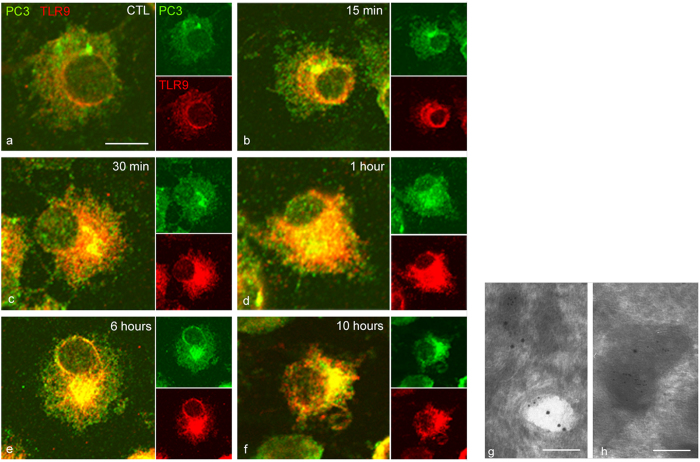
PC1/3 and TLR9 translocate to the same vesicular structures immediately upon exposure of cells to CpG-ODN. Confocal images of NR8383 cells double-labelled with anti-PC1/3 (green, Alexa488) and anti-TLR9 (red, Alexa546). (**a**) Untreated cells. (**b–f′**) Cells incubated with 100 nM CpG-ODN 2006 for the indicated durations. All of the confocal images are shown at the same scale, bar = 10 μm. (**g,h**) Immunogold electron micrographs, showing the coexistence of PC1/3 (small gold particles, 6 nM) and TLR9 (large gold particles, 12 nM) in the same endosome (**g**) and in the same lysosome (**h**) after incubation with 100 nM CpG-ODN 2006 for 6 h. Bar = 250 nM for both electron micrographs.

**Figure 5:**
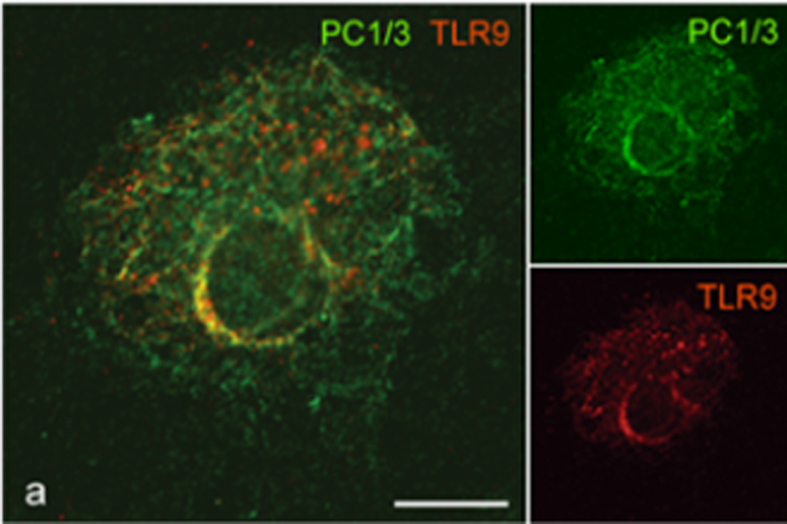
Bafilomycin A1 completely blocked PC1/3-TLR9 co-localization induced by CpG-ODN stimulation. NR8383 cells were stimulated for 6 h with 100 nM CpG-ODN 2006 in the presence of 100 nM bafilomycin A1, which was added to the cells 2 h prior to stimulation. The cells were then double labelled using anti-PC1/3 and anti-TLR9 and imaged by confocal microscopy. Bar = 10 μm.

We performed time-lapse confocal microscopy to examine the spatial behaviours of PC1/3 and TLR9 after treatment with 100 nM CpG-ODN 2006 (Fig. 4a–f). In resting cells, we found that PC1/3 and TLR9 did not co-localize (Fig. 4a). However, after only 15 min of CpG-ODN stimulation, a portion of TLR9 was detected in a PC1/3-positive compartment (Fig. 4b). After 30 min and 1 h of incubation, the areas of co-localization extended progressively throughout the cytoplasm and toward the cell periphery (Fig. 4c,d). After longer incubation times (6 and 10 h), the co-localization signal became very strong and similar distributions of PC1/3 and TLR9 were observed (Fig. 4e–f′). To better characterize the exact organelle to which PC1/3 and TLR9 co-localized after stimulation, we conducted immunoelectron microscopic analysis. Double immunogold labelling clearly highlighted that PC1/3 and TLR9 coexisted in the same endosomes and lysosomes of treated NR8383 cells (Fig. 4g,h). Our results showed a correlation between TLR9 and PC1/3 activations (Fig. 2). TLR9 is cleaved to give rise to its active form in endosomes with a low pH and it is known that PC1/3 activity also requires an acidic pH. Therefore, the co-localization observed between the receptor and the proprotein convertase may occur in endosomes with low pH. To test this hypothesis, we examined the effect of bafilomycin A1 (bafA1) a compound which blocks endosomal acidification by inhibiting the intravesicular hydrogen pumps V-ATPase. After pre-incubation with bafA1 during 2 hours, NR8383 cells were exposed to CpG-ODN during 6 hours and examined by confocal microscopy. We found that PC1/3 did not co-localize anymore with TLR9 except in the ER (Fig. 5) demonstrating that PC1/3 and TLR 9 co-localized in endosomes with acidic pH. Thus, PC1/3 likely controls TLR9 cleavage and activation.

### PC1/3 does not control TLR9 activation by proteolytic cleavage

**Figure 6:**
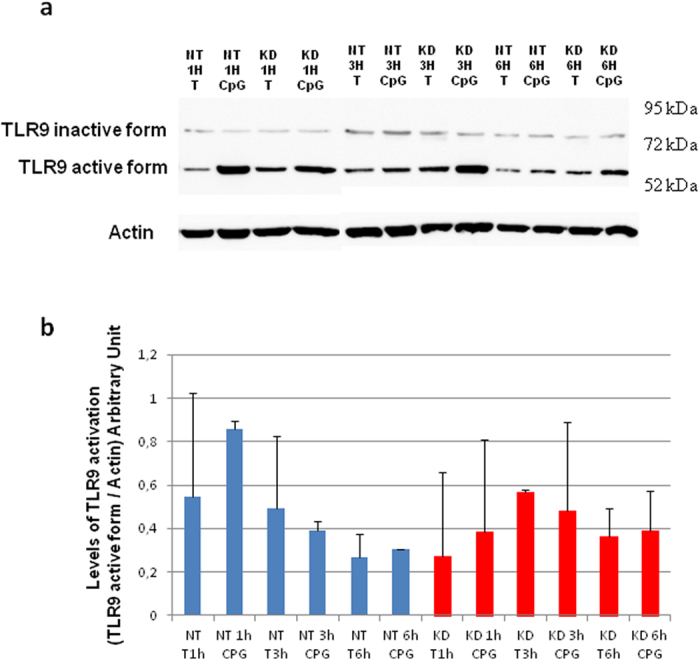
PC1/3 does not control TLR9 activation by proteolytic cleavage. Non-target (NT) and PC1/3 knockdown (KD) NR8383 cells were exposed to 1 μM CpG-ODN 2006 for 1, 3 or 6 h. (**a**) Proteins were then extracted, and western blotting was carried out with anti-TLR9. Intensities of 64 kDa bands corresponding to TLR9 active form were quantified and normalized to that of actin. (**b**) The results are depicted through graphic representations. Experiments were performed in triplicate and the data are presented as the mean ± SD. Data were then analyzed by three ways ANOVA followed by Holm-Sidak posthoc test.

To clarify if PC1/3 may control TLR9 activation, we took advantage of a PC1/3-knockdown (KD) NR8383 cell line obtained after lentiviral shRNA delivery^19^. Since TLR9 activation is directly linked to its spatiotemporal compartmentalization, Non-Target shRNA (NT) and PC1/3 KD NR8383 cells were challenged with CpG-ODN during 1 h, 3 h and 6 h. TLR9 proteolytic cleavage was then studied by western blot (Fig. 6a). Intensity of the 64 kDa band, corresponding to TLR9 active form (Fig. 2b), was quantified. However, after time course experiments, no differences were registered between NT and KD cells as revealed by three ways ANOVA followed by Holm-Sidak posthoc test (Fig. 6b). This demonstrates that PC1/3 doesn’t control TLR9 cleavage and thus its activation.

### PC1/3 regulates TLR9 trafficking

**Figure 7:**
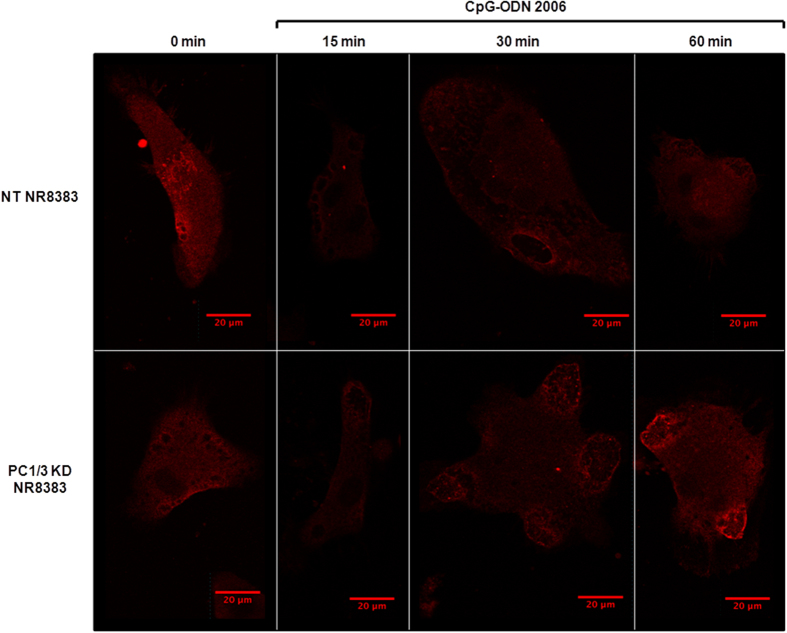
PC1/3 regulates TLR9 localization. Non-target (NT) and PC1/3 knockdown (KD) NR8383 cells were exposed to 100 nM CpG-ODN 2006 for 0, 15, 30 or 60 min, labelled with anti-TLR9 (red) and analyzed by confocal microscopy.

**Figure 8:**
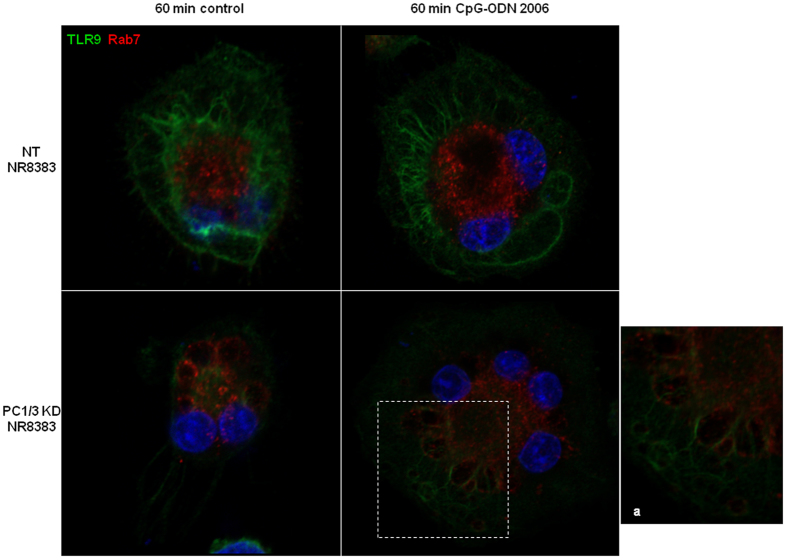
In the absence of PC1/3, TLR9 clusterizes in multivesicular bodies (MVB) after treatment with CpG-ODN. NT and PC1/3 KD NR8383 cells were exposed to CpG-ODN 2006 for 60 min and stained with anti-TLR9 (green) and anti-Rab7 (red). The nuclei were counterstained with Hoechst 33342 (blue). Confocal microscopy analysis was then performed. A higher magnification of the enclosed region is shown in (**a**). It points out the co-localization of TLR9 and Rab7 demonstrating the cluterization of TLR9 in MVB.

We next tested if PC1/3 may regulate TLR9 trafficking. Confocal imaging data of NT and PC1/3 KD cells in time course revealed that under CpG-ODN treatments TLR9 formed aggregates which were much more intense in KD cells (Fig. 7). Labelling with Rab7 indicated that TLR9 clusterization in KD cells occured in multivesicular bodies (Fig. 8). This demonstrates that PC1/3 is crucial for the proper trafficking of TLR9. In this context, we speculated that in PC1/3 KD cells, the targeting of TLR9 to MVBs may differentially trigger the associated signaling pathways. To address this hypothesis, we investigated both STAT3 and NF-κB activation.

### Under CpG-ODN treatment, NF-κB activation occurs rapidly in PC1/3 KD cells

**Figure 9:**
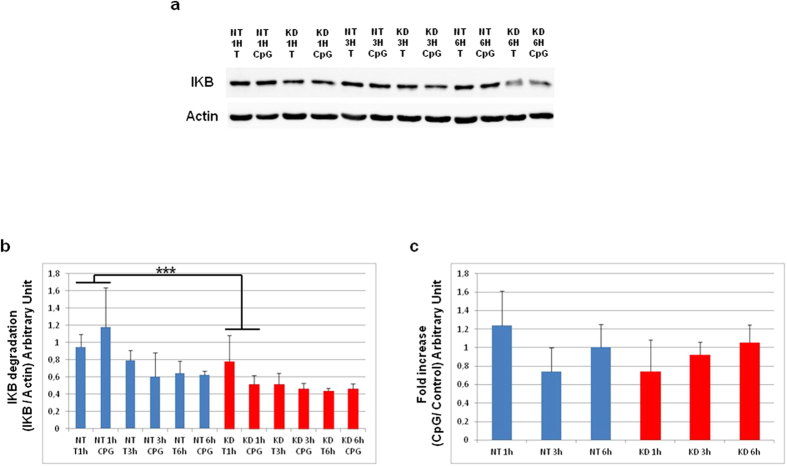
Time course of IκB-α degradation after CpG-ODN treatment. (**a**) Western blot analysis of total IκB-α in NT or KD PC1/3 NR8383 macrophages treated with 1 μM CpG-ODN 2006 for 1, 3 and 6 h. Intensities of total IκB-α bands were quantified and normalized to that of actin. (**b**) The results are depicted through graphic representations (**c**). The fold increases in the samples stimulated with CpG-ODN relative to the non-stimulated samples are shown. Experiments were performed in triplicate and data were then analyzed by two (**c**) or three (**b**) ways ANOVA followed by Holm-Sidak posthoc test. ***Significant differences between NT cells and KD cells (p < 0.001).

To examine NF-kB activation, we analyzed by western blot the degradation of IκB-α after CpG-ODN treatment for 1, 3, or 6 h (Fig. 9a). Data were then analyzed by three ways ANOVA followed by Holm-Sidak posthoc test. This revealed that IκB-α level was significantly lower at 1h (p < 0.001) in PC1/3 KD cells compared to NT cells (Fig. 9b). We next studied the fold increases between (Fig. 9c) CpG-ODN challenged cells vs. control cells to follow the dynamics of IκB-α degradation and synthesis. Despite a non-significant two ways ANOVA followed by Holm-Sidak posthoc test, the IκB-α level displayed a tendency to be lower in KD cells than in NT cells at 1 h after challenge. This reflected a more intense and quicker degradation of IκB-α and thus an increased activation of NF-κB in KD cells. At 3 h, we observed the opposite situation and at 6 h, both cell types had the same IκB-α protein level. Taken together, these results show that the NF-κB pathway is less repressed and more rapidly activated in KD cells. These effects may be the consequence of the clustering of TLR9 in MVBs, as observed in KD cells (Fig. 8).

### STAT3 signaling is repressed in PC1/3 KD cells

**Figure 10:**
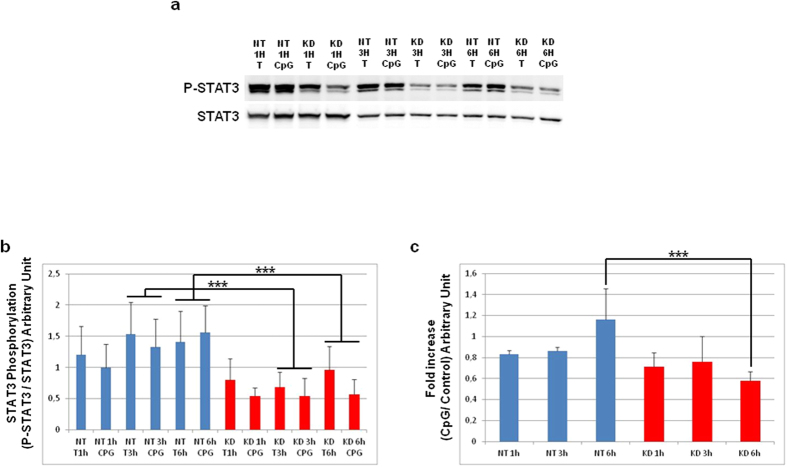
STAT3 signaling is repressed in PC1/3 KD cells. (**a**) Western blot analysis of phospho-STAT3 in NT or KD PC1/3 NR8383 macrophages treated with 1 μM CpG-ODN 2006 for 1, 3 and 6 h. Intensities of phospho-STAT3 bands were quantified and normalized to that of total STAT3. (**b**) The results are depicted through graphic representations. (**c**) The fold increases in the samples stimulated with CpG-ODN relative to the non-stimulated samples are shown. Experiments were performed in triplicate and data were then analyzed by two (**c**) or three (**b**) ways ANOVA followed by Holm-Sidak posthoc test. ***Significant differences between NT cells and KD cells (p < 0.001).

As observed in Fig. 9, PC1/3 downregulation promoted the pro-inflammatory pathway via NF-κB activation. To address whether PC1/3 inhibition also favoured pro-inflammatory phenotype through repression of the anti-inflammatory pathway, we focused on STAT3 activation. Indeed, STAT3 has been described as a negative feedback inhibitor of TLR9 signalling^21^. To assess STAT3 activation, we analyzed by western blot the phosphorylation state of STAT3 after CpG-ODN treatment for 1, 3, or 6 h (Fig. 10a). Data were then analyzed by three ways ANOVA followed by Holm-Sidak posthoc test. This revealed that the level of phosphorylated STAT3 was significantly lower at 3 h and 6 h (p < 0.001) in PC1/3 KD cells than in NT cells (Fig. 10b). Differences in the dynamics of STAT3 phosphorylation under CpG-ODN challenge were also observed according to the fold changes in the CpG-ODN vs. control cells. Indeed, a two ways ANOVA followed by Holm-Sidak posthoc test pointed out a significant decrease of STAT3 phosphorylation in KD cells at 6 h post-treatment (p < 0.001, Fig. 10c). These data indicate that, in the presence or absence of CpG-ODN treatment, STAT3 pathway activation is reduced in KD cells.

### After CpG-ODN treatment, PC1/3 KD cells secrete more pro-inflammatory cytokines than NT cells

**Figure 11:**
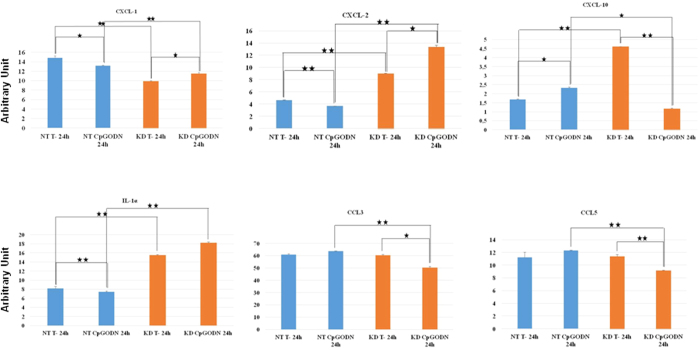
Cytokines secreted by NT and KD cells during challenge (or not) with CpG-ODN. Cells were untreated (control) or treated with 1 μM CpG-ODN for 24 h. A Rat Cytokine Array was used to probe cytokines in the secretomes. The NT cell secretomes are shown in blue and the KD cell secretomes are depicted in orange. The bar diagrams show the ratios of the spot mean pixel densities/reference point pixel densities. Significant differences were analyzed using Student’s t-test. *P ≤ 0.05, **P ≤ 0.01.

To correlate TLR9 signaling and trafficking with cytokine secretion, cytokine arrays were performed after CpG-ODN treatment of NT and PC1/3 KD cells. At 4 h post-treatment, no significant differences in cytokine secretion were observed between NT and PC1/3 KD cells (data not shown). At 24 h post-treatment, the CXCL1, CXCL2, CXCL10, CCL3, CCL5 and IL1α levels were found to be altered (Fig. 11). The CXCL2 and IL1α levels were significantly increased in KD cells compared with those in NT cells after stimulation. The CXCL10 level was increased in KD cells compared with that in NT cells under basal condition and it dropped following the CpG-ODN treatment. In untreated and treated conditions, the CCL3 level in KD cells was lower than in NT cells. Finally, we observed that the CCL3 and CCL5 levels decreased in KD cells compared with those in NT cells after stimulation.

## Discussion

In tumors, macrophages tend to acquire the anti-inflammatory M2 phenotype and to promote cancer growth by suppressing immune cell function^1^. Therefore, strategies need to be developed to reactivate macrophages. In this context, understanding how Toll-like receptors (TLRs) are activated, how they traffic and trigger their signaling pathways is crucial. Recently, studies have focused on TLR9 because it is considered as a potent therapeutic target for the effective treatment of some cancers^24^. TLR9 activation by CpG-DNA depends on its spatiotemporal compartmentalization in the endolysosomal compartment, where it undergoes specific proteolytic cleavage by cathepsins and asparagine endopeptidases^25^,^26^,^27^,^28^,^29^. Recently, we have demonstrated that the key endoprotease, proprotein convertase 1/3 (PC1/3) regulates intracellular trafficking machinery, the endosomal pathway and TLR4 activity^17^. We suspected that PC1/3 may also control TLR9 trafficking and activity. In neuroendocrine cells, PC1/3 activation requires specific proteolytic cleavages, which occurs during PC1/3 trafficking from the endoplasmic reticulum (ER) to secretory granules^18^,^19^. Here, we have demonstrated for the first time in macrophages that PC1/3 matures and co-traffics with TLR9 to the endolysosomal system after CpG-ODN treatment (Figs 2, 3, 4). In contrast, we have observed that when PC1/3 is inhibited, TLR9 targeting is modified and the receptor forms aggregates in multi vesicular bodies (MVBs) (Fig. 8). However, TLR9 cleavage is not impaired by PC1/3 inhibition (Fig. 6). These results are in contrast with those reported for the activity of another proprotein convertase family member, furin, in the TLR7 antiviral immune response^30^. In fact, furin is not required for TLR7 trafficking but is necessary for its proteolytic cleavage at a neutral pH, possibly in early endosomes or in a pre-endosomal compartment such as the ER, Golgi, or plasma membrane^30^. This finding is consistent with the reported activity of proprotein convertases as endoproteases. Taken together, our data suggest a new role for proprotein convertases as key molecules involved in TLR trafficking. Recently, a crucial role of neurite outgrowth inhibitory protein isoform B (NOGO-B) in proper TLR9 trafficking to the endolysosomal system has been demonstrated in macrophages^31^. Notably, we have previously observed that compared with NT cells, PC1/3 KD cells exclusively secrete Nogo^17^. Therefore, PC1/3 may control TLR9 targeting by mediating Nogo-B expression. We have also previously described overexpression of proteins involved in protein trafficking between the trans-Golgi network (TGN) and endosomes in PC1/3 KD cells^17^. Further, we have shown that the cytoskeleton is substantially reorganized following PC1/3 inhibition^17^. Therefore, another hypothesis is that PC1/3 may regulate TLR9 trafficking through regulation of trafficking machinery and cytoskeletal reorganization. This activity is similar to what occurs during UNC93B1-mediated recruitment of adaptor protein complex 2 (AP-2) for TLR9 delivery to endolysosomes^32^. It is also comparable to what happens during control of CpG-induced tubular endolysosomal extension and proper distribution of TLR9 by Ly49Q^16^. Thus, the precise mechanism exerted by PC1/3 to regulate TLR9 trafficking remains to be determined. However, we have showed here that the redistribution of TLR9 in MVBs in PC1/3 KD cells impacts the associated signalling pathways (Figs 9 and 10) and resulting cytokines secretion (Figs 1 and 11). Indeed, the NF-κB pro-inflammatory pathway was more rapidly and strongly activated (Fig. 9). This finding is supported by the fact that the anti-inflammatory cytokine IL1-Ra was not secreted by PC1/3 KD cells after challenge (data not shown). This is in contrast with what has been observed in wild type mouse macrophages after CpG-ODN stimulation^33^. We also depicted that PC1/3 inhibition in CpG-ODN-stimulated macrophages resulted in induction of secretion of pro-inflammatory cytokines, i.e., TNFα and IL-6 in PC1/3 KO mice (Fig. 1) and IL1α and CXCL2 in PC1/3 KD rat NR8383 (Fig. 11). Interestingly, we have previously demonstrated that the pro-inflammatory transcription factor STAT1 is co-expressed with NF-κB1 in LPS-stimulated KD cells, whereas this activity does not occur in LPS-stimulated NT cells^17^. KD cells also spontaneously secrete CXCL10^17^, a cytokine whose gene expression is controlled by STAT1. Moreover, in myeloid cells, type I interferons activate STAT1 and STAT3^34^. STAT1 is sequestered by STAT3 to prevent STAT1 homodimer formation, thereby suppressing STAT1 activity. STAT3 has also been described to be a negative feedback inhibitor of TLR9 signaling in hematopoietic cells. Indeed, ablating *Stat3* in these cells results in rapid activation of innate immunity by CpG, with enhanced production of IFN-γ, TNFα and IL-12^21^. Our present study revealed that STAT3 activity was repressed in PC1/3 KD cells and that this repression was increased under CpG-ODN treatment (Fig. 10). We have also showed that after CpG-ODN challenge, the initially high level of CXCL10 also known as an interferon gamma-inducer, markedly dropped. It indicates that PC1/3 inhibition skews TLR9 signalling towards the NF-κB pathway instead of the IRF pathway as we have shown for TLR4. Taken together, these findings show that inhibition of PC1/3 orients macrophages toward a pro-inflammatory phenotype. TLR9^35^, NF-κB and STAT3^20^,^21^ are key molecules involved in tumor propagation. Therefore, control of their activation by PC1/3 is a promising strategy to reactivate tumor-associated macrophages and to develop a potent antitumor therapy.

## Materials and Methods

### Reagents

The rat alveolar macrophage NR8383 cell line (CRL-2192) was obtained from ATCC (USA). The rabbit anti-PC1/3 (Fus) antibody used has been previously described and characterized^18^,^19^. Mouse monoclonal anti-TLR9 (26C593.2) was purchased from NOVUS Biologicals. Rabbit anti-Rab7, mouse anti-IκB-α, rabbit anti-STAT3, rabbit anti-phospho-STAT3 and mouse anti-Actin were acquired from Cell Signaling Technology. Alexa Fluor® 488 donkey anti-rabbit, Alexa Fluor® 488 donkey anti-mouse, Alexa Fluor® 555 donkey anti-rabbit and Alexa Fluor® 555 donkey anti-mouse secondary antibodies were obtained from Molecular Probes. Phosphorothioate CpG-ODN 2006 was acquired from Invivogen. Ham’s F12K medium, puromycin, phosphate-buffered saline (PBS), and fetal bovine serum (FBS) were purchased from Life Technologies (Milan, Italy). Nitrocellulose membranes and a Bio-Rad Protein Assay Kit were purchased from Bio-Rad (Marnes La Coquette, France). SuperSignal West Dura Chemiluminescent Substrate was acquired from Thermo Scientific. A peroxidase-conjugated secondary antibody was obtained from Jackson ImmunoResearch (West Grove, PA, USA). Rat Cytokine Array Panel A was purchased from R&D Systems (Minneapolis, MN, USA).

### Mouse Experimental Models

The transgenic PC1/3 and wild-type (WT) mice used in this study were between 3 and 6 months of age. The mice were maintained in a pathogen-free environment and were provided food and water *ad libitum*. PC1/3 KO mice have been previously described^18^. They were generated by deletion of exon 1 and several upstream transcriptional control elements of the PCSK1 gene by inserting a neomycin cassette in the C57BL/6 mouse background. All experimental protocol were approved by the Canadian Council on Animal Care and licensing committee from sherbrooke University. The methods were carried out in accordance with the approved guidelines.

### Determination of Cytokine Secretion and Cellular Content of Primary Peritoneal Macrophages

Mice were injected intraperitoneally with 2 ml of sterile 3% thioglycolate (BD Biosciences) to increase the yield of peritoneal macrophages. Three days later, the mice were anesthetized with ketamine/xylazine (87/13 mg/kg intramuscularly) and were then sacrificed by cervical dislocation. Peritoneal cells were collected by peritoneal wash with a phosphate-buffered saline (PBS) solution. Red blood cells were lysed by incubation with hemolysis buffer. Cells were plated in a 6 or 24 well plate at 8 × 10^5^ or 2.4 × 10^6^ cells, respectively. They were cultured in RPMI 1640 medium containing penicillin/streptomycin for 24 h at 37 °C in a humidified atmosphere with 5% CO_2_ in air. This allowed macrophage adherence, resulting in purification of the peritoneal exudates. The following day, the medium was replaced by serum free medium and the cells were stimulated for 4 h with PBS 1X or 1 μM CpG-ODN. Medium was collected and a cell lysate was obtained by adding 200 μl of 0.5 N HCl followed by three freeze-thaw cycles. Total protein was collected by centrifugation and cytokine levels were measured using ELISA kits specific for mouse TNFα and Mouse IL-6 according to the manufacturer’s instructions (R&D Systems). Statistical analysis was performed using Student’s t test with Prism 5 (GraphPad Software), which calculates the SE.

### Culture of NR8383 cell lines

Rat alveolar wild-type (WT) NR8383 macrophages were cultured in Ham’s F12K medium supplemented with 15% fetal bovine serum. NR8383 PC1/3 knockdown (PC1/3-KO) and NR8383 non-target (NT) shRNA cell lines were cultured in Ham’s F12K medium supplemented with 15% fetal bovine serum and 12 μg/ml puromycin. NR8383 PC1/3 knockdown was carried out using lentiviral transduction, as described previously^19^. Culture was performed at 37 °C in a humidified atmosphere (5% CO2).

### Confocal microscopy and co-localization studies

The NR8383 cells were grown on cover slip and treated or not as described. The cells were then fixed with 4% paraformaldehyde (PFA) for 10 min. After washes with PBS, cells were permeabilized with 0.2% Triton X-100 for 10 min at room temperature and blocked during 1 h with a blocking solution (PBS 1X, 1% Normal Donkey Serum, 1% BSA, 0.01% Triton). Cells were then incubated overnight at 4 °C with the primary antibodies diluted at 1:100 in blocking solution. After intensive washes with PBS 1X, secondary antibodies diluted at 10 μg/mL in blocking solution was applied for 1 h. After washes with PBS, the nuclei were stained with Hoechst 33342 (1/10000) and the cells were visualized by confocal microscopy. Fluorescence analysis was conducted using a Zeiss LSM 510 confocal microscope (488 nm excitation for Alexa 488 and 543 nm for Alexa 546) connected to a Zeiss Axiovert 200 M with a 63 × 1.4 numerical aperture oil immersion objective. Both channels were excited, collected separately and then merged to examine the co-localization. The image acquisition characteristics (pinhole aperture, laser intensity, scan speed) were the same throughout the experiments to ensure comparability of the results.

### Measurement of Texas red-dextran uptake

Endocytic activity was assessed by incubating cells for 2 h with 0.5 mg/ml Texas Red-dextran at 37 °C. Cells were washed extensively with PBS 1X and fixed with 4% paraformaldehyde (PFA) for 10 min. PC1/3 labelling with anti-PC1/3 was then performed as described above.

### Bafilomycin A1 Treatment

NR8383 cells were stimulated for 6 hours with 100 nM CpG-ODN in the presence of 100 nM bafilomycin A1 added to the cells 2 hours prior to stimulation. The cells were then double labelled using anti-PC1/3 and anti-TLR9 and imaged by confocal microscopy.

### Electron microscopy

After incubation or not with 100 nM CpG-ODN 2006, cells were harvested by scraping and the medium was removed by centrifugation. For the morphological experiments, cell pellets were fixed in 2.5% glutaraldehyde in 0.1 M cacodylate buffer, pH 7.4, for 15 min on ice. After washes, cell pellets were postfixed in 1% osmium tetroxide in the same buffer for another 15 min at room temperature. The pellets were subsequently processed for embedding in Epon resin after dehydration in graded acetonitrile. Double-contrasted sections (uranyl acetate and lead citrate^36^) were observed under a Hitachi H600 transmission electron microscope set at 75 kV. For ultrastructural immunodetection, cell pellets were fixed in 4% paraformaldehyde and 0.1% glutaraldehyde in PBS 1X for 15 min on ice. After several washes in PBS 1X, the pellets were infused overnight in 2.3 M sucrose and 20% polyvinyl pyrrolidone. The pellets were then frozen and kept in liquid nitrogen until use. Cryosections were harvested on Formvar-coated grids^37^. The grids were then incubated in PBS-glycerol (50 mM, pH 7.4) for 20 min at room temperature to inactivate the residual aldehydes. Next, they were transferred to a blocking solution (PBS, 5% activated BSA-C, 5% donkey serum albumin, and 0.1% fish gelatin) for 30 min at room temperature. After several washes, the samples were incubated with the respective primary antibodies for either 1 h at room temperature or overnight at 4 °C in a moist chamber. After thorough washes, the grids were incubated with their respective secondary antibodies labelled with colloidal gold (6 and 12 nM) for 1 h at 37 °C. The sections were finally contrasted with uranyl acetate and embedded in methyl cellulose^37^.

### Total protein extraction

NR8383 PC1/3-KD and NT cells were plated on sterile 6 well plates and cultured until they reached confluence. For CpG-ODN stimulation, cells were starved overnight in Ham’s F12K medium supplemented with 2% FBS. They were then stimulated with 1 μM CpG-ODN 2006 in serum-free medium or were left untreated. At 24 h, cells were collected, washed once with ice-cold PBS and then lysed with RIPA buffer for total protein extraction (150 mM NaCl, 50 mM Tris, 5 mM EGTA, 2 mM EDTA, 100 mM NaF, 10 mM sodium pyrophosphate, 1% NP40, 1 mM PMSF, and 1X protease inhibitors). Cell debris was removed by centrifugation (20000 g, 10 min, 4 °C), supernatants were collected and protein concentrations were measured using a Bio-Rad Protein Assay Kit, according to the manufacturer’s instructions.

### Organelles sub-fractionation

8 to 9 × 10^7^ cells were harvested and processed for subcellular fractionation. The pellet was homogenized in 1 ml Tris 10 mM, sucrose 0.25 M, pH 7.5 and antiprotease cocktail, on ice in a Kontes glass homogenizer fitted with a 15 μm clearance pestle. The success of the homogenization process was checked by phase contrast microscopy (at least 90% broken cells). After two centrifugations at 800 g in order to remove the nuclei and the cellular debris, the post nuclear supernatant was laid on top of a preformed (two chamber gradient maker, CBS. Scientific) continuous (10–30%) gradient of Iodixanol (Optiprep™, Axis-Shield) and spun for 1H30 at 60,000 g in a swinging bucket rotor (SW41Ti, Beckman). The gradient was then collected from the bottom in 1 ml fractions which were 3 fold diluted in homogenization medium and centrifuged for 30 min in a fixed angle rotor (50Ti, Beckman) at 220,000 g. Each fraction was dissolved in Laemmli buffer and then processed through SDS-PAGE (8% acrylamide).

### Western Blot Analysis

Total cell extracts (40 μg) were then analyzed by Western blotting. First, proteins were separated by SDS-PAGE electrophoresis and transferred onto nitrocellulose membranes. The membranes were blocked for 1 h at room temperature with TBS-Tween 0.1% + 5% nonfat dry milk and incubated overnight at 4 °C with primary antibodies against TLR9 (1:1000), IκBα (1:1000), phosphor-STAT3 (1:2000), STAT3 (1:1000) and actin (1:1000). The membranes were washed with TBS-Tween 0.1%, incubated for 1 h at room temperature with peroxidase-conjugated secondary antibodies (anti-mouse and anti-rabbit diluted at 1:30000 or 1:20000, respectively) and washed again with TBS-Tween 0.1%. Proteins were visualized with an enhanced chemiluminescence kit (West Dura from Pierce) according to the manufacturer’s instructions. ImageJ software was used to quantify the bands.

Data were then analyzed by two or three ways ANOVA followed by Holm-Sidak posthoc test.

### Identification of cytokines using rat cytokine antibody arrays

NR8383 PC1/3-KD and NT cells were plated on sterile 6 well plates and cultured until they reached confluence. The cells were starved overnight in Ham’s F12K medium supplemented with 2% FBS and were stimulated for 4 h or 24 h with 1 μM CpG-ODN or were left untreated. Cell supernatants were collected, centrifuged at 500 g, passed through a 0.22 μm filter to remove cells and immediately frozen in liquid nitrogen. A Rat Cytokine Array Panel A (R&D Systems) was used to probe cytokines in the secretomes of stimulated and unstimulated NR8383 cells according to the manufacturer’s recommendations. Briefly, array membranes were first incubated in blocking buffer for 1 h. In the meantime, secreted proteins were mixed with Detection Antibody Cocktail and incubated for 1 h at room temperature. The secreted protein volume used for this experiment was determined according to the cell count after stimulation. Next, after removal of the blocking buffer, sample/antibody mixtures were added to array membranes and incubated overnight at 4 °C. After incubation, the membranes were washed 3 times with Wash Buffer and then incubated with a streptavidin-HRP solution for 30 min at room temperature. The membranes were finally washed 3 times with Wash Buffer and bound antibodies were detected by chemiluminescence using Chemi Reagent Mix. The membranes were quantified by densitometry using ImageJ software. Statistical analysis was carried out by Student’s t test.

## Additional Information

**How to cite this article**: Duhamel, M. *et al*. The proprotein convertase PC1/3 regulates TLR9 trafficking and the associated signaling pathways. *Sci. Rep.* **6**, 19360; doi: 10.1038/srep19360 (2016).

## Supplementary Material

Supplementary Information
