# Supplementary material for: The proprotein convertase PC1/3 regulates TLR9 trafficking and the associated signaling pathways
Source: Sci Rep. 2016 Jan 18;6:19360. doi: 10.1038/srep19360 (PMC4725977; doi:10.1038/srep19360)
Supplement: Supplementary Information [file srep19360-s1.pdf]

The proprotein convertase PC1/3 regulates TLR9 trafficking and the associated signaling pathways.

Duhamel M, Rodet F, Murgoci A.N., Desjardins R, Gagnon H, Wisztorski M, Fournier I, Day R, Salzet M

Duhamel M, Rodet F, Murgoci A.N., Desjardins R, Gagnon H, Wisztorski M, Fournier I, Day R, Salzet M

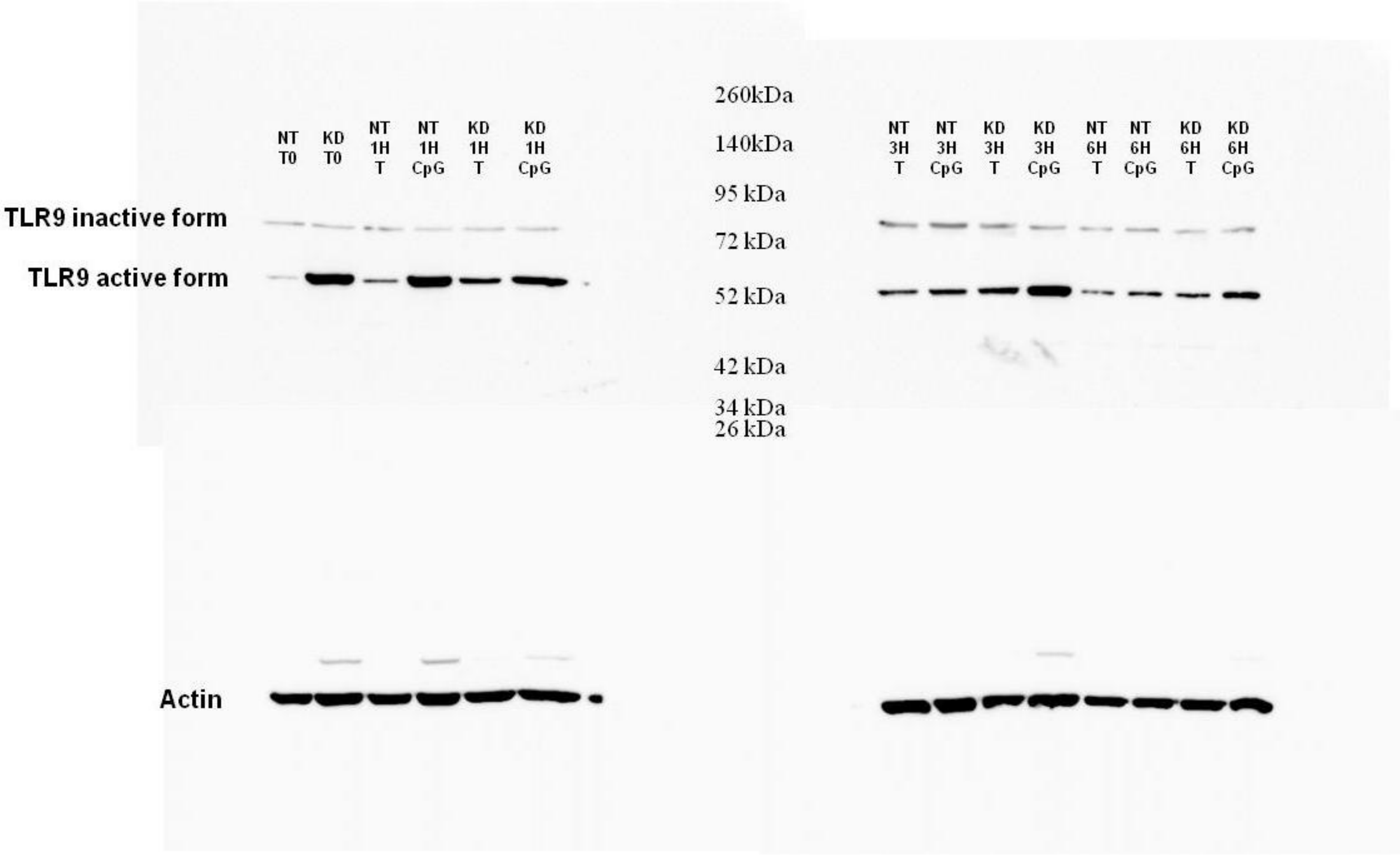

<u>**Supplementary Material 1:**</u> **Unprocessed original scans for the blots in figure 6 PC1/3 does not control TLR9 activation by proteolytic cleavage.**
Non-target (NT) and PC1/3 knockdown (KD) NR8383 cells were exposed to 1 µM CpG-ODN 2006 for 0, 1, 3 or 6 h. Proteins were then extracted, and western blotting was carried out with anti-TLR9. To assess that an equal amount of proteins was loaded on gels, membrane was stripped and reprobed with anti-Actin.

Duhamel M, Rodet F, Murgoci A.N., Desjardins R, Gagnon H, Wisztorski M, Fournier I, Day R, Salzet M

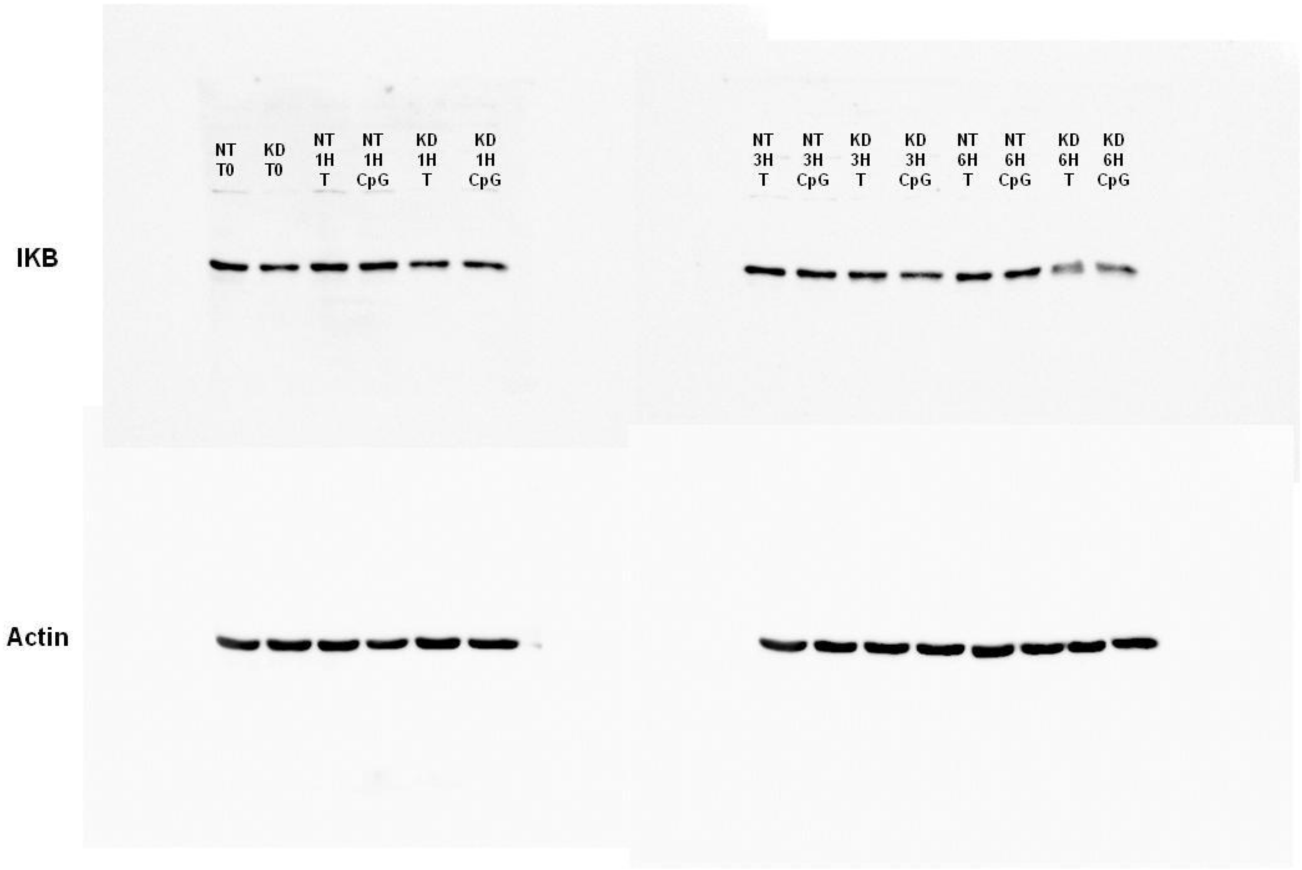

Supplementary Material 2: Unprocessed original scans for the blots in figure 8 Time course of IκB-α degradation after CpG-ODN treatment.
Western blot analysis of total IκB-α in NT or KD PC1/3 NR8383 macrophages treated with 1 µM CpG-ODN 2006 for 1, 3 and 6 h. To assess that an equal amount of proteins was loaded on gels, membrane was stripped and reprobed with anti-Actin.

Duhamel M, Rodet F, Murgoci A.N., Desjardins R, Gagnon H, Wisztorski M, Fournier I, Day R, Salzet M

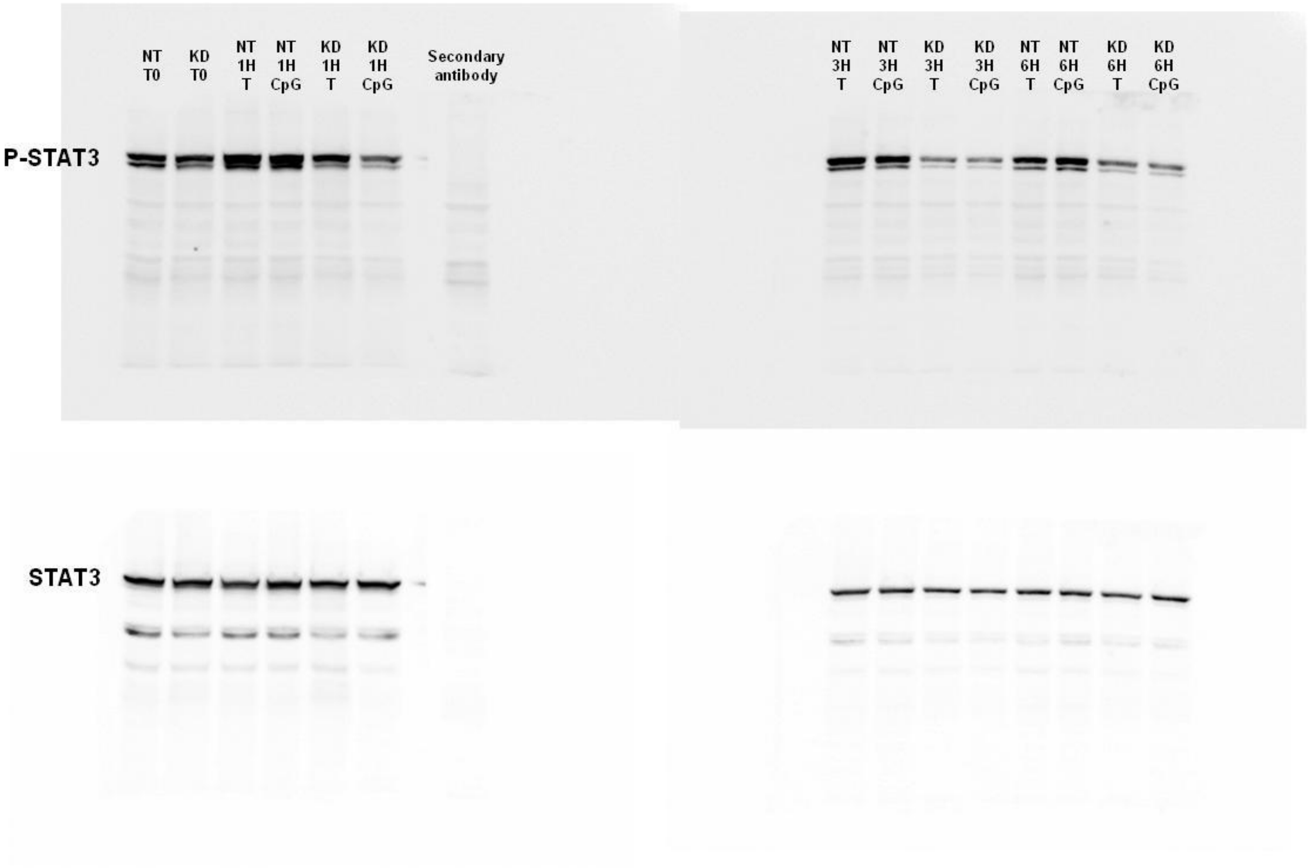

<u>Supplementary Material 3:</u> Unprocessed original scans for the blots in figure 9 STAT3 signaling is repressed in PC1/3 KD cells

Western blot analysis of phospho-STAT3 in NT or KD PC1/3 NR8383 macrophages treated with 1 µM CpG-ODN 2006 for 1, 3 and 6 h. As a control, a membrane was incubated with the secondary antibody alone. To assess that an equal amount of proteins was loaded on gels, membrane was stripped and reprobed with anti- total STAT3.
